# Substrate temperature dependence of structure and optical properties of ZnTiO_3_:Er^3+^/Yb^3+^ thin films synthesized by pulsed laser deposition

**DOI:** 10.1016/j.heliyon.2023.e16259

**Published:** 2023-05-12

**Authors:** S.J. Mofokeng, F.V. Molefe, R.E. Kroon, H.C. Swart, T.P. Mokoena, M.S. Dhlamini, M.J. Sithole, L.L. Noto

**Affiliations:** a Department of Physics, College of Science Engineering and Technology, University of South Africa, Johannesburg, 1710, South Africa; b Department of Physics, Tshwane University of Technology, Private Bag X680, Pretoria, 0001, South Africa; c Department of Physics, University of the Free State, Bloemfontein, ZA9300, South Africa

**Keywords:** Er^3+^/Yb^3+^ co-doped zinc titanate, ZnTiO_3_ XPS, Up-conversion mechanism, Cross relaxation, Photons

## Abstract

ZnTiO_3_:Er^3+^,Yb^3+^ thin film phosphors were successfully deposited by pulsed laser deposition (PLD) at different substrate temperatures. The distribution of the ions in the films was investigated and the chemical analysis showed that the doping ions were homogeneously distributed in the thin films. The optical response of the phosphors revealed that the reflectance percentages of the ZnTiO_3_:Er^3+^,Yb^3+^ vary with the silicon substrate temperature due to the differences in the thickness and morphological roughness of the thin films. Under 980 nm diode laser excitation, the ZnTiO_3_:Er^3+^,Yb^3+^ film phosphors displayed up-conversion emission from the Er^3+^ electronic transitions, with violet, blue, green, and red emission lines at 410, 480, 525, 545 and 660 nm from ^2^H_9/2_ → ^4^I_15/2_, ^4^F_7/2_ → ^4^I_15/2_, ^2^H_11/2_ → ^4^I_15/2_, ^4^S_3/2_ → ^4^I_15/2_ and ^4^F_9/2_ → ^4^I_15/2_ transitions, respectively. The up-conversion emission was enhanced by increasing the silico (Si) substrate temperature during the deposition. Based on the photoluminescence properties and decay lifetime analysis, the energy level diagram was established and the up-conversion energy-transfer mechanism was discussed in detail.

## 1 Introduction

The development of nanocrystalline semiconducting thin films has become a topic of interest in the field of research, because of their many characteristic properties and diverse applications [1]. In the past years, research has been carried out on the application of metal oxide semiconductor thin films on photovoltaic (PV) cells. They used either pure metal oxide or doped semiconductors with down-conversion (DC) and up-conversion (UC) rare-earth ions in the host lattice to enhance the efficiency of various PV cells [[2], [3], [4]]. Research has been carried out to study the effects of the optical properties of oxide compounds such as ZnTiO_3_ [2,5], Ta_2_O_5_: Eu, Ce [6], SiO_2_–TiO_2_:Er^3+^/Yb^3+^/Eu^3+^ [7], CeO_2_:Er, Yb [8], etc., on the efficiency of PV cells. Among these oxides, ZnTiO_3_ displays good catalytic properties with different polymorphs, and it is widely used in the PV cell field [9].

ZnTiO_3_ is a ternary oxide that has been proven to enhance the PV response, due to its larger electron mobility and wide band gap. There are different phases of zinc titanate that crystallize at different temperatures, which include Zn_2_Ti_3_O_8_, ZnTiO_3_ and Zn_2_TiO_4_, just to mention a few. The Zn_2_Ti_3_O_8_ is a metastable phase, which crystallizes at temperatures lower than 800 °C. ZnTiO_3_ is thermodynamically stable between 800 and 850 °C, and Zn_2_TiO_4_ crystallizes at temperatures of 850 °C and above. ZnTiO_3_ resembles a rhombohedral ilmenite structure [10,11]. Sahu et al. [10] have investigated the evolution of a different crystallographic phase of zinc titanate annealed at different temperatures for PV applications. When ZnTiO_3_ is coated on PV cells, it absorbs certain wavelengths of the electromagnetic spectrum. To improve the efficiency of the PV process, the coating needs to be doped with UC rare earth ions that convert photons that would not be absorbed by the cell to absorbable photons, like converting near infrared (NIR) photons to visible photon emission [9].

The use of rare earth co-doped oxide materials to adapt the incident spectrum of sunlight can increase the number of absorbable incident photons and enhance the power conversion efficiency of a PV cells. The well-known rare earth ions for UC from the infrared bands to the visible band include Er^3+^, Tm^3+^, Ho^3+^, Pr^3+^ and Nd^3+^. These ions are normally selected as activators which generate UC luminescence for 980 nm radiation. However, one of the drawbacks of the above activators is the low luminescence intensity due to their low absorption cross-sections for 4f–4f transitions. Recently, there are several techniques that are adopted to enhance the UC emission intensity, such as pairing UC activator with a sensitizer and changing the crystal phase of the host materials. An interesting ion is Yb^3+^, because it has proven to be an effective sensitizer in generating efficient UC luminescence when co-doped with UC activators by subsequently transfer their harvested energy to neighbouring UC activator ions [[12], [13], [14], [15]]. The Yb^3+^ ion is very interesting because of its strong absorption at around 980 nm excitation, low non-radiative decay, and simple electronic structure consisting of only two manifolds, ^2^F_7/2_ and ^2^F_5/2_ manifolds separated by approximately 10,000 cm^−1^ [16,17].

In this work, ZnTiO_3_ co-doped with Er^3+^ and Yb^3+^ phosphor was synthesized via the conventional solid-state reaction synthesis method. The phosphor was coated as thin films on Si(100) substrates at different temperatures using the PLD technique. The study was carried out to investigate the effects of the different substrate temperatures on the luminescence properties of these films.

## 2 Experimental details

### 2.1 Experimental procedure

ZnTiO_3_:Er^3+^,Yb^3+^ phosphor was prepared by the conventional solid-state reaction technique. Commercial zinc oxide (ZnO, Sigma-Aldrich, purity 99.9%), titanium dioxide (TiO_2_, Sigma-Aldrich, purity 99.7%), erbium (III) acetate hydrate (Er(CH_3_COO)_3_·H_2_O, Sigma-Aldrich, purity 99.9%) and ytterbium (III) acetate hydrate (Yb(CH_3_COO)_3_·H_2_O, Sigma-Aldrich, purity 99.9%) were used as starting materials. The stoichiometric amounts of ZnO and TiO_2_ were first mixed and ground together in a ball mill for 1 h at room temperature. To optimize the samples, different molar concentrations (0.2–1.2 mol%) of Er^3+^ were incorporated into the ball milled powder and mixed using a pestle and mortar. The same method was used for Yb^3+^ co-doping at various molar concentrations (0.1–0.9 mol%). The obtained powder, 0.8 mol% of erbium acetate and 0.7 mol% of ytterbium acetate were mixed all together using pestle and mortar. The phosphor powders were further calcined in air, at 800 °C for 3 h.

The ZnTiO_3_:Er^3+^,Yb^3+^ thin films were prepared using the PLD technique. The phosphor powder was pressed using an in house-built hydraulic press to prepare the ablation target. The resulting target was annealed at 800 °C for 8 h in air, and then mounted on the rotating target holder into the PLD system. The films were grown on Si(100) substrates. The Si substrates were cleaned inside an ultrasonic bath using ethanol for 15 min, dried by blowing nitrogen (N_2_) gas on their surfaces and mounted on the substrate holder which was inserted parallel to the target inside the PLD system. The distance between the Si substrate and the target was kept constant at 3.5 cm. The PLD chamber was pumped down to 3.00 × 10^−5^ mbar, and then backfilled with oxygen (O_2_) to a partial pressure of 13 mbar. The 266 nm fourth harmonic of a Nd:YAG laser (8 ns, 40 mJ/pulse, 0.767 J/cm^2^ and 10 Hz pulse repetition rate) was used for the target evaporation and the deposition time was 30 min for all the samples. The substrate temperature was varied from 100 to 600 °C, with interval of 100 °C.

### 2.2 Characterization techniques

The crystalline structure of the thin films was examined using an X-ray diffractometer (XRD) Rigaku Smartlab that was equipped with a monochromatic CuK α (λ = 0.15405 nm) irradiation source that was operated at 200 mA current and 45 kV. The particle surface morphology and the elemental composition of the thin films were studied by using a Jeol JSM-7800 field emission scanning electron microscope (FE-SEM) coupled with an Oxford X-Max80 Energy X-ray Dispersive Spectroscopy (EDS) detector. The distribution of the ions on the surface and the 3D thin film maps were obtained using the Time-of-Flight secondary ion mass spectrometer (IONTOF TOF-SIMS^5^) with a spatial resolution of 512 × 512 pixels. A pulsed 30 kV Bi^+^ primary ion beam operated at a DC current of 0.4 pA and pulse repetition rate of 10 kHz (100 μs), was used to probe the surface. For the acquisition of depth profiles, the O_2_^+^ sputter gun operated at 1 kV and 250 nA was used. The analysis and sputter areas were 100 × 100 μm^2^ and 300 × 300 μm^2^, respectively. The chemical composition was conducted using a PHI 5000 Versaprobe-scanning ESCA X-ray photoelectron spectroscopy (XPS) system. The diffuse reflectance (DR) studies of the powders were evaluated with a Perkin Elmer Lambda 1050 UV–Vis–NIR absorption spectrometer. The photoluminescence (PL) measurements were conducted using an Edinburgh Instruments FLS980 system with a 980 nm (2 W) diode laser as an excitation source for UC luminescence. UC luminescence was recorded by a Hamamatsu R928P photomultiplier tube, which was operated in photon counting mode after passing through a double emission monochromator. The colour coordinates were calculated by Commission Internationale de L’Eclairage (CIE) software (GoCIE) to confirm the colour tuning behavior of the emitted light.

## 3 Results and discussion

### 3.1 Structural and morphological analysis of thin film samples

**Fig. 1:**
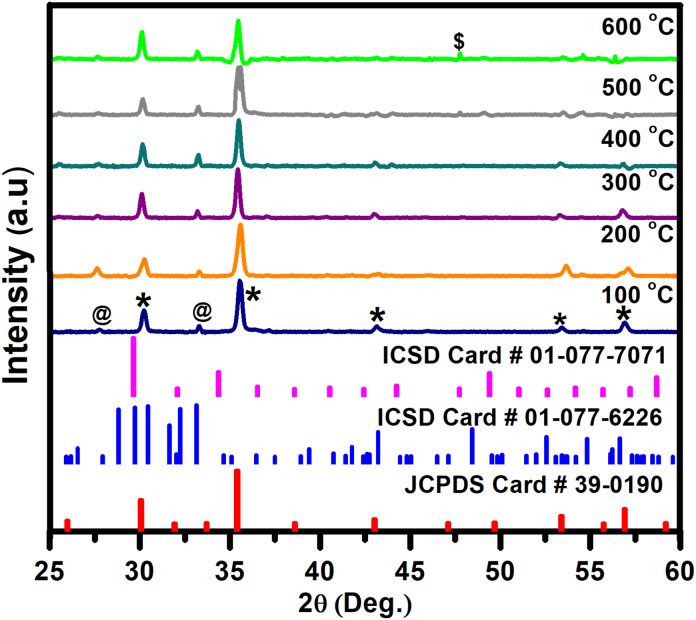
X-ray diffraction patterns of the ZnTiO_3_: Er^3+^,Yb^3+^ thin films deposited on the Si(100) which was heated at different temperatures. The peaks marked by an asterisk * correspond to ZnTiO_3_, @ correspond to Er_2_O_3_ and $ correspond to Yb_2_O_3_.

Fig. 1 depicts the XRD patterns of the thin films deposited on the Si(100), which were heated at different temperatures. Similar XRD peaks were obtained under all different substrate temperature and this is an indication of the successful ZnTiO_3_: Er^3+^,Yb^3+^ thin film fabrication. The diffraction peaks in the XRD patterns of ZnTiO_3_ occur at angles that agree well with the Joint Committee on Powder Diffraction Standard (JCPDS) card number 39-0190. The XRD results revealed the presence of secondary phases of Er_2_O_3_ (ICSD file no. 01-077-6226) and Yb_2_O_3_ (ICSD file no. 01-07-7071). All the diffraction peaks of ZnTiO_3_ compound can be indexed to the (2 2 0), (3 1 1), (4 0 0), (4 2 1), and (5 1 1) planes at *2θ* = ∼30.2°, 35.5°, 40.4°, 53.4°, and 56.9°, respectively. Minor diffraction peaks of the secondary phases corresponding to Er_2_O_3_ observed at ∼27.8° and ∼33.3° can be indexed to the (−1 1 1) and (−1 1 2) planes, respectively. A single diffraction peak of the secondary peaks associated to Yb_2_O_3_ at higher substrate temperature is observed at ∼47.8° can be indexed to the (1 2 5). The formation of Er_2_O_3_ and Yb_2_O_3_ phases might be due to doping concentrations as reported in our previous studies [18].

**Fig. 2:**
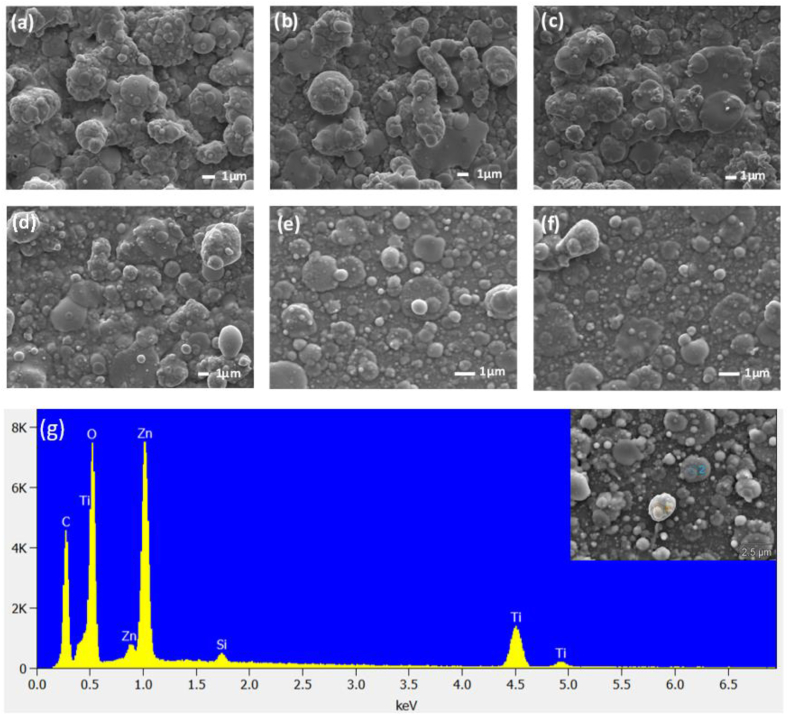
Surface SEM images of ZnTiO_3_: Er^3+^,Yb^3+^ thin films fabricated at various substrate temperatures of (a) 100 °C, (b) 200 °C, (c) 300 °C, (d) 400 °C, (e) 500 °C, and (f) 600 °C and the corresponding (g) EDS spectrum of ZnTiO_3_: Er^3+^,Yb^3+^.

Fig. 2 presents the surface morphologies of the ZnTiO_3_: Er^3+^,Yb^3+^ films deposited at different temperatures. The particles sizes on the surface decreased with an increase of substrate temperatures. Fig. 2(a)–(c) depict that the surface morphologies of the thin films fabricated at a substrate temperature range of 100–400 °C showed large grains mixed with fine grains. For a substrate temperature of 400 °C and above, the obtained thin films shown in Fig. 2(d)–(f) show that the particles became spherical, and uniformly residing on irregular shapes. The slight change in morphology at higher substrate temperatures can be attributed to the nucleation process of ZnTiO_3_: Er^3+^,Yb^3+^ compound onto the surface of the Si substrate due to higher deposition temperature [19]. The elemental composition was determined using EDS spectroscopy. As displayed in Fig. 2(g), the characteristic peaks of Zn, Ti and O can be prominently discerned, confirming the presence of ZnTiO_3_. Furthermore, Si can be detected, as befits silicon substrate. The peaks due to Yb and Er ions were not detected by EDS due to low dopant concentrations or because EDS spectroscopy was insufficiently sensitive to detect Yb and Er in the prepared films [20,21]. XPS, on the other hand, detected Yb and Er due to its surface sensitivity to elements [22].

**Fig. 3:**
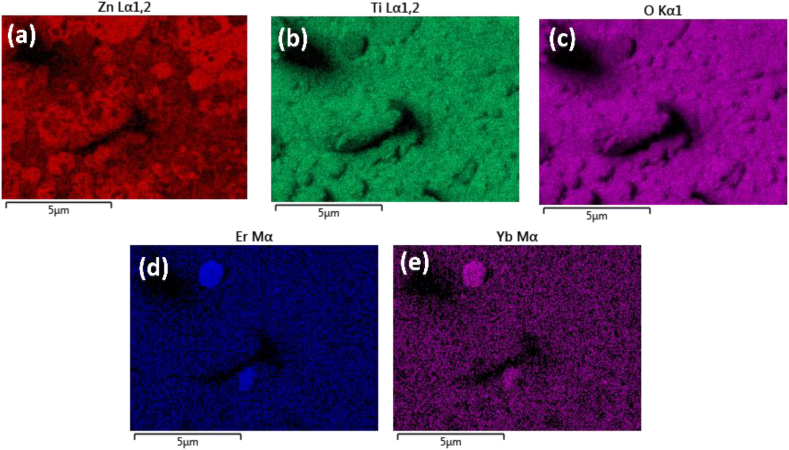
EDS X-ray mapping of the elements in ZnTiO_3_: Er^3+^,Yb^3+^ thin film deposited at 500 °C.

Fig. 3 depicts the EDS mapping for the elements in ZnTiO_3_: Er^3+^,Yb^3+^ thin film fabricated at a temperature of 500 °C, since luminescence measurements shows that this was the optimum substrate temperature with the strongest emission intensity. For this reason we have decided to perform EDS mapping, analytical and chemical characterization of this thin film. The mapping confirms that the surface of ZnTiO_3_:Er,Yb thin film consisted of Zn, Ti, O, Er and Yb elements, with no other elements found on the surface of the film. Furthermore, the elemental mapping in Fig. 2(d) and (e) also revealed a uniform distribution of the Er and Yb dopant ions in the compound of the deposited film. These observations correspond to the observed ZnTiO_3_, Er_2_O_3_ and Yb_2_O_3_ crystalline phases during XRD analysis [23,24].

### 3.2 TOF-SIMS analysis of thin film samples

**Fig. 4:**
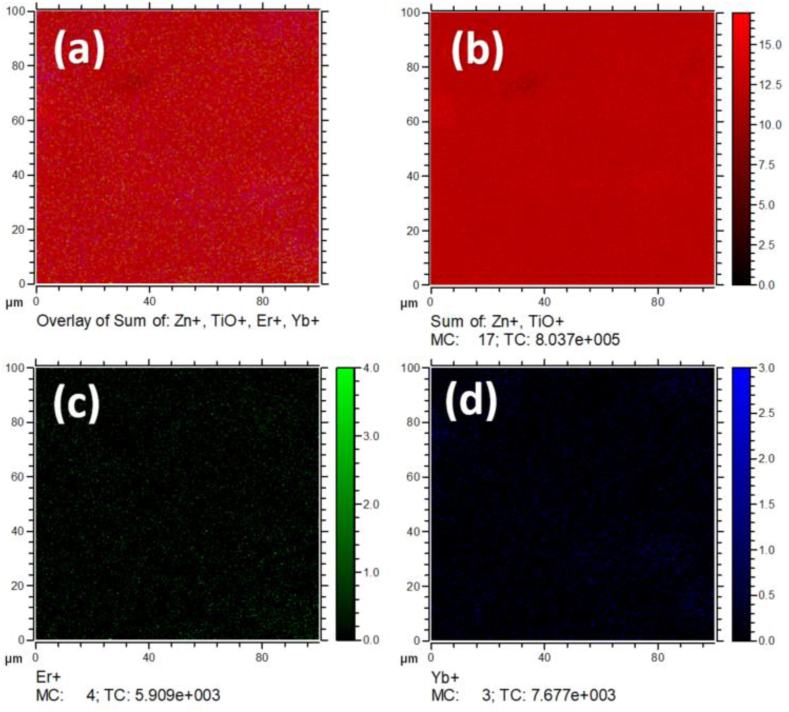
A TOF-SIMS overlay chemical image of 0.8 mol% Er^3+^ and 0.7 mol% Yb^3+^ co-doped ZnTiO_3_ thin film on a Si(100) substrate temperature of 500 °C.

Fig. 4 depicts the distribution of the Er^3+^ and Yb^3+^ ions in ZnTiO_3_ phosphor which was investigated using the TOF-SIMS analysis. The surface mapping of the film was obtained over an area of 100 × 100 μm^2^. The surface distribution of the ions was acquired from the optimum sample because after the deposition of the films, the homogeneity of the distribution of the ions in the films is not changing. Fig. 4(a) depicts the image overlay mapping of the thin film. The distribution of the ions on the surface of the films is shown in different colours for the overlay mapping to allow comparison of all ions at once. The sum of Zn^+^ and TiO^+^ ions are presented as red (Fig. 4 (b)), Er^+^ ions as green (Fig. 4 (c)), and Yb^+^ ions as blue (Fig. 4 (d)). The overlay images revealed the homogenous distribution of the Zn^+^, TiO^+^, Er^+^ and Yb^+^ ions in this region, indicating a successful incorporation in the phosphor sample [25].

**Fig. 5:**
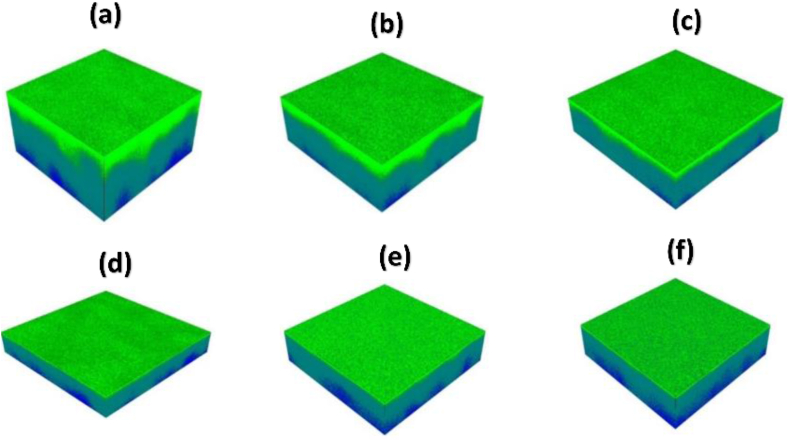
3D overlay chemical composition of ZnTiO_3_: Er^3+^,Yb^3+^ thin film prepared at different substrate temperature (a) 100 °C, (b) 200 °C, (c) 300 °C, (d) 400 °C, (e) 500 °C and (f) 600 °C.

Fig. 5 depicts a 3D overlay analysis of the ZnTiO_3_:Er^3+^,Yb^3+^ samples and the Si substrate at different substrate temperatures. The green colour represents the Ti^+^ ions and the blue colour represent the Si^+^ ions of the substrate while the inter-diffusion process is represented by the cyan colour. The images show that the heating allowed the inter-diffusion process of deposited material and substrate. It was also observed that the inter-diffusion process can be locally enriched by increasing the substrate temperature or heat treatment, as also reported by Balakrishna et al. [26].

**Fig. 6:**
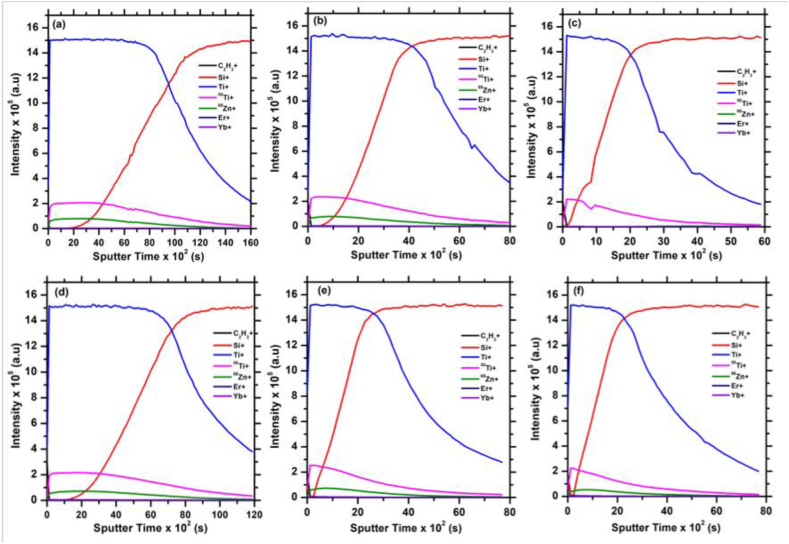
TOF-SIMS depth profiles of the ZnTiO_3_: Er^3+^,Yb^3+^ thin film deposited at different substrate temperatures (a) 100 °C, (b) 200 °C, (c) 300 °C, (d) 400 °C, (e) 500 °C and (f) 600 °C.

The TOF-SIMS depth profiles (Fig. 6) for ZnTiO_3_: Er^3+^,Yb^3+^ films were collected in order to investigate the elemental distribution with depths from the surface down to the substrate of the thin films. They were recorded in a positive secondary ion mode by the Bi^+^ ion beam. The Ti^+^ and Si^+^ signals are of great interest in all depth profile spectra shown in Fig. 6, as they reveal the layers that are readily detected. The ^68^Zn^+^ was taken to monitor the Zn in the samples and the relative sensitivity factor for Zn^+^ is very small/low compared to Ti^+^ and Si^+^. The figures shows that the ZnTiO_3_:Er^3+^,Yb^3+^ films consist of two distinct regions, one which is predominantly the thin film material and the other enriched with Si^+^ ions. The two regions in this case reflect the inter-diffusion of Si at the ZnTiO_3_/Si interface during the deposition at different temperatures [27,28]. The C_2_H_3_^+^, ^50^Ti^+^, ^68^Zn^+^, Er^+^ and Yb^+^ signals are almost constant with an increase in substrate temperature. In both overlay images (Fig. 5) and depth profile (Fig. 6), the films produced at lower substrate temperature appeared to be thicker compared to other films and become thinner with an increase of the substrate temperature.

### 3.3 Chemical states of the thin film samples

**Fig. 7:**
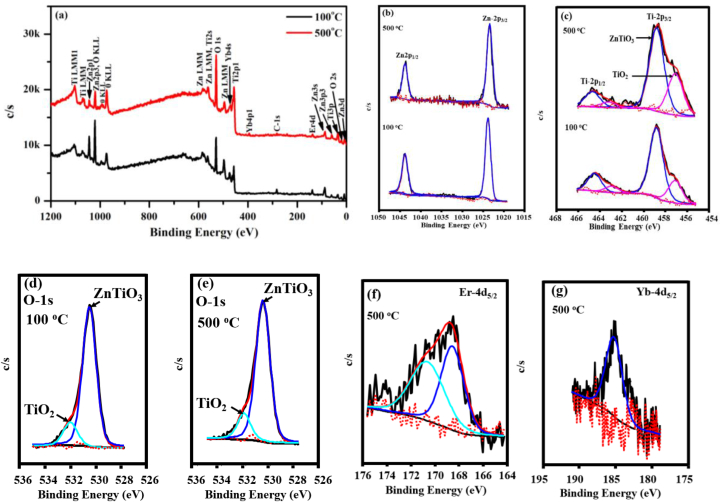
(a) XPS survey scan of ZnTiO_3_: Er^3+^,Yb^3+^ thin films. High resolution XPS spectra of ZnTiO_3_: Er^3+^,Yb^3+^ thin film for (b) Zn-2p region, (c) Ti-2p region, (d)–(e) O-1s core levels, (f) Er-4d region and (g) Yb-4d region.

To investigate the chemical and electronic states of the elements within ZnTiO_3_:Er, Yb thin films, an XPS PHI 5000 versaprobe with 100 m, 25 W, and 15 kV Al monochromatic x-ray beam was used. The spectrometer was manufactured by Physical Electronics (PHI) which is a division of ULVAC-PHI. The excitation source was a focused electron beam scanned onto an Al anode for X-ray generation and a quartz crystal monochromator that focuses and scans the generated X-ray beam onto the sample surface at a power of 25 W. The spectra were collected for 100 ms per step at 0.1 eV intervals. The pass energy was set to 11 eV, which resulted in an analyzer resolution of ≤0.5 eV. The monochromator was based on a 200 mm Rowland circle with quartz (100) crystals on an ellipsoidal substrate to generate micro focused X-ray beam. The sputtering etching was carried out with an Ar Ion gun (2 kV 2 μA) at a 1 × 1 mm Raster Sputter rate of approximately 12 nm/min. The charge referencing was C 1s–284.8 eV. Fig. 7 depicts the XPS survey scan and the high resolution (HR) scans used to evaluate the surface chemical state of ZnTiO_3_: Er^3+^,Yb^3+^ thin films deposited on the substrate temperature at 100 °C and 500 °C. XPS measurements were performed on the 100 °C and 500 °C samples for comparison between the optimum sample and the thin film fabricated at lowest substrate temperature. Fig. 7(a) shows full survey scan spectrum of the thin films. The presence of Zn, Ti, O, Yb, and Er elements is clearly visible in the figure, correlating with observations from XRD and EDS mapping data. The high HR spectra of Zn-2p are shown in Fig. 7(b). The Zn-2p spectrum is split into two peaks, the Zn-2p_3/2_ and Zn2p_1/2_ with energy difference of 22.97 eV [29]. The peaks of the samples prepared at 100 and 500 °C were fitted with single peak, corresponding to a typical Zn^2+^ in the oxygen-deficiency lattice structure of the ZnTiO_3_ [[30], [31], [32]]. The XPS quantification of ZnTiO_3_:Er, Yb thin films is presented in Table 1. Fig. 7(c) depicts the HR spectra of Ti-2p core level, and the samples exhibited the major two peaks, which are attributed to spin-orbit of Ti-2p_3/2_ and Ti-2p_1/2_ doublets. The Ti signal at 100 °C and 500 °C were also split into two peaks, the Ti-2p_3/2_ and Ti-2p_1/2_ which were fitted with two peaks, corresponding to Ti from ZnTiO_3_ [33] and Ti from TiO_2_ [34]. The TiO_2_ contribution was from the unreacted reagent during the preparation of ZnTiO_3_. This observation is in line with the XRD data (Fig. 1). It was reported that the chemical reaction of ZnO and TiO_2_ occurs in the sample and the crystallization of the production of ZnTiO_3_ species is due to oxidation of Ti during the chemical reaction [31,34]. However, Fig. 7(c) revealed that at lower substrate temperatures, the unreacted TiO_2_ was greater, and this suggests that at higher substrate temperatures, the thin film is likely to crystallize into more than one phase. Fig. 7(d) and (e) shows the core-level XPS spectra of O-1s state of ZnTiO_3_:Er, Yb thin films. The O-1s core level was deconvoluted into two peaks positioned at 530.5 and 531.9 eV, which are a contribution from ZnTiO_3_ and TiO_2_, respectively. The intense O-1s peak, which is positioned at 530.5 relative to O-1s, which is positioned at 531.9 peak in Fig. 7(d) and (e), suggest a spatial distribution of Zn^2+^ ions across the surface of TiO_2_ species [31]. Fig. 7(f) shows the Er-4d characteristic peaks of ZnTiO_3_: Er^3+^,Yb^3+^ thin film. The Er-4d_5/2_ located at 168.80 and 170.90 eV, belong to the signals of Er_2_O_3_ and Er_2_(C_2_O_4_)_3_ species, respectively [35]. The detection of Er_2_(C_2_O_4_)_3_ species on the ZnTiO_3_ surface might originated from the chemical reaction between erbium (III) acetate hydrate and zinc oxide/titanium dioxide precursors used during the synthesis process [36]. In addition, Yb-4d_5/2_ characteristic peak located at 185.4 eV as shown in Fig. 7(g) is assigned to Yb_2_O_3_ [37].

**Table 1:** XPS quantitative and oxidation states of ZnTiO_3_:Er, Yb thin films.

Peak name	Temperature Compound distribution	Peak position (^o^C)	Chi-square		Oxidation state	% Area
			(eV)	$\left(\chi^{2}\right)$		
Zn	100	1022.20	3.35	2p_3/2_	66.10	ZnTiO_3_
		1045.20	3.35	2p_1/2_	33.90	ZnTiO_3_
Zn	500	1022.30	1.18	2p_3/2_	68.20	ZnTiO_3_
		1045.20	1.18	2p_1/2_	32.00	ZnTiO_3_
Ti	100	457.30	1.43	2p_3/2_	19.00	TiO_2_
		458.80	1.43	2p_1/2_	60.01	ZnTiO_3_
		463.00	1.43	2p_3/2_	5.50	TiO_2_
		464.40	1.43	2p_1/2_	15.40	ZnTiO_3_
Ti	500	457.34	1.38	2p_3/2_	29.00	TiO_2_
		458.90	1.38	2p_1/2_	56.25	ZnTiO_3_
		462.95	1.38	2p_3/2_	3.92	TiO_2_
		464.28	1.38	2p_1/2_	10.82	ZnTiO_3_
O	100	530.50	1.37	1s	84.30	ZnTiO_3_
		531.90	1.37	1s	15.70	TiO_2_
O	500	530.50	1.92	1s	84.30	ZnTiO_3_
		531.90	1.92	1s	15.80	TiO_2_
Er	500	168.80	0.62	4d_5/2_	65.70	Er_2_O_3_
		170.90	0.62	4d_5/2_	34.30	Er_2_(C_2_O_4_)_3_
Yb	500	185.77	0.65	4d_5/2_	100.0	Yb_2_O_3_

### 3.4 Diffuse reflectance of the thin film samples

**Fig. 8:**
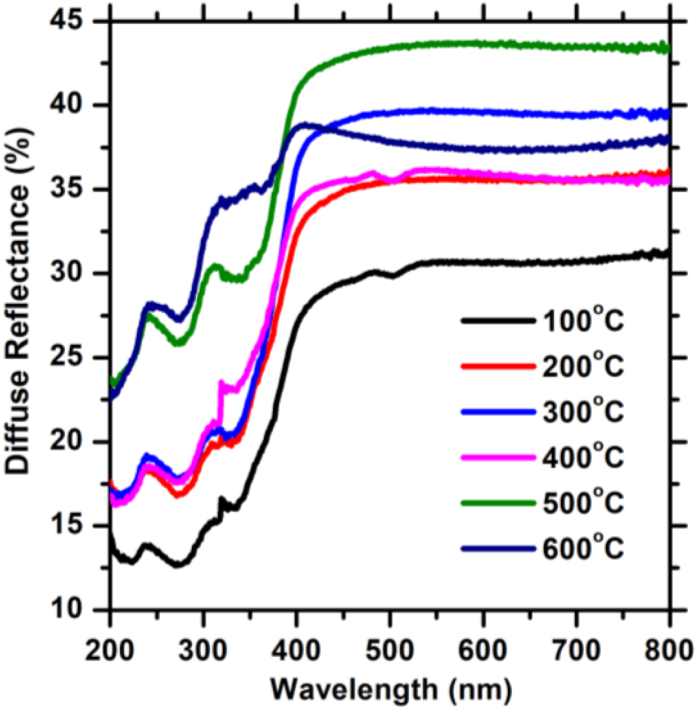
The diffuse reflectance spectra of ZnTiO_3_: Er^3+^,Yb^3+^ thin films grown at different substrate temperatures.

The diffuse reflectance spectra of ZnTiO_3_:Er^3+^,Yb^3+^ thin films deposited at different temperatures were recorded, as shown in Fig. 8. The samples show low reflectance (25–45%) in the visible spectral region (i.e., 380–700 nm) and 12–35% reflectance in the UV region. In addition, the region between 330 and 400 nm corresponds to the absorption edge of ZnTiO_3_. The reflectance of the samples varies with the substrate temperature due to the differences in the thickness and morphological roughness of the films [38]. The observed bands below 330 nm in the reflectance spectra are an indication of additional absorbing centres within the conduction band of the material, which may be attributed to the deposition effects, because it was not present in the phosphor powders as reported in our previous communication [18]. As a result, the reflectance spectra show that the prepared thin films have a better absorption of visible electromagnetic waves, compared to the ZnTiO_3_:Er^3+^,Yb^3+^ phosphor powders [18].

### 3.5 UC luminescence analysis

**Fig. 9:**
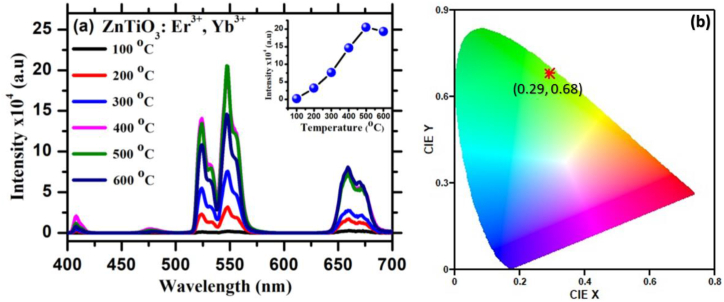
(a) UC emission spectra of ZnTiO_3_:Er^3+^,Yb^3+^ thin film at different substrate temperature from 100 to 600 °C and (b) CIE chromaticity coordinates of ZnTiO_3_:Er^3+^,Yb^3+^ thin film at substrate temperature of 500 °C.

Fig. (a) depicts the visible emission spectra of ZnTiO_3_:Er^3+^,Yb^3+^ thin films at different substrate temperatures, which were measured after exciting the samples with a 980 nm diode laser. The spectra show five emission peaks at 410, 480, 525, 547 and 660 nm, assigned to ^2^H_9/2_ → ^4^I_15/2_, ^4^F_7/2_ → ^4^I_15/2_, ^2^H_11/2_ → ^4^I_15/2_, ^4^S_3/2_ → ^4^I_15/2_ and ^4^F_9/2_ → ^4^I_15/2_ electronic transitions of Er^3+^ ions, respectively [1,19,[39], [40], [41]]. The emission intensity was increased with an increase in substrate synthesis temperature and then decreased after reaching the maximum intensity as shown in the inset of Fig. 9(a). The emission intensity increased with increasing substrate synthesis temperature and then decreased after reaching the maximum intensity due to luminescence concentration quenching, as shown in the inset of Fig. 9(a), due to the presence of non-radiative energy transfer processes between active Er^3+^ and Yb^3+^ ions at higher substrate temperature [39]. The optimum substrate temperature which is at 500 °C, shows the strongest UC emission intensity. This points out that the growth of ZnTiO_3_:0.8Er^3+^,0.7 Yb^3+^ thin films on the substrate temperature of 500 °C is an optimum condition with intense UC luminescence emission of Er^3+^ and Yb^3+^ ions. According to Jafer et al. [42] and Elleuch et al. [19], the increase in luminescence emission intensity with the increase in the substrate temperature is due to the improvement of the crystal quality and particle morphology of the ZnTiO_3_:Er^3+^,Yb^3+^ thin film at higher substrate temperature. Fig. 9(b) depicts the Commission Internationale de l'Eclairage (CIE) colour coordinates employed in order to evaluate the dominant characteristic emission colour of the optimized ZnTiO_3_:Er^3+^,Yb^3+^ thin film. The CIE chromaticity coordinate of the optimized ZnTiO_3_:Er^3+^,Yb^3+^ thin film is (0.29, 0.68).

**Fig. 10:**
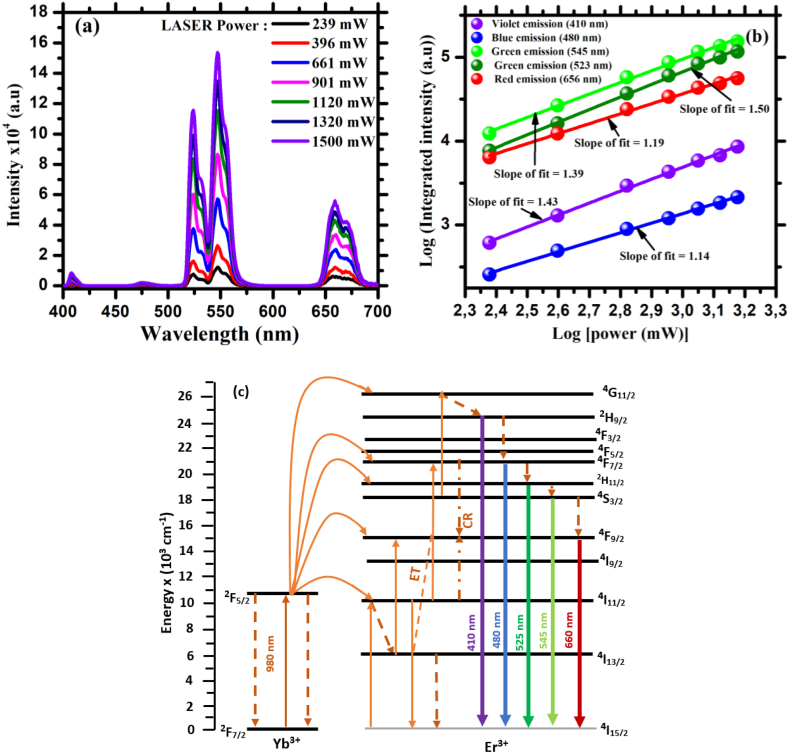
(a) UC emission spectra with variation of laser power from 239 mW to 1500 mW, (b) logarithmic dependence of emission intensity as a function of logarithm of power for ZnTiO_3_:Er^3+^,Yb^3+^ thin film at a substrate temperature of 500 °C and (c) Energy transfer mechanism showing the radiative transitions for UC process in ZnTiO_3_:Er^3+^,Yb^3+^ thin films under an excitation of 980 nm.

Fig. 10(a) shows the emission spectra with the variation of laser power for the ZnTiO_3_:Er^3+^,Yb^3+^ thin film at a substrate temperature of 500 °C. It can be seen from the figure that the emission intensity of the sample was increased with an increase in laser power. The pumping power dependence spectra of the UC luminescence intensity for ZnTiO_3_:Er^3+^,Yb^3+^ thin film under 980 nm laser excitation was investigated and presented in Fig. 9(b). The minimum number of pumping photons involved in the mechanism of up-conversion in ZnTiO_3_:Er^3+^,Yb^3+^ thin film may be estimated from the power dependency spectra, which can be represented as equation (Eq. (1)) [43]

(1) log(I) = nlog(P)

where *I* denote the integrated luminescence intensity of UC luminescence, *n* is the number of photons, and *P* is the laser power. The observed slope values of the linear fit of the log-log power dependences for violet, blue, green and red emissions are 1.43, 1.14, 1.39, 1.50, and 1.19. The observed slope values suggest that a two-photon process is expected for the UC luminescence properties of the ZnTiO_3_:Er^3+^,Yb^3+^ thin film. According to the literature, the UP progress of violet emission is typically associated with three-photon absorption photons [[44], [45], [46], [47]]. However, the slope of this emission band at 410 nm in the produced ZnTiO_3_:Er^3+^,Yb^3+^ thin film is 1.43, implying that the UC process of this emission band should be a two-photon absorption process. This can be explained in terms of the influence of Yb^3+^ ions doping concentration, where a high concentration of Yb^3+^ doping in the optimized ZnTiO_3_:Er_3+_,Yb^3+^ sample is expected to create a significant number of Er^3+^ ions at the ^4^I_11/2_ energy state. In this regard, energy transfer between two neighbouring Er^3+^ ions is projected to occur due to Er^3+^ ions lingering on the ^4^I_11/2_ energy level for a longer period of time. In this context, Er^3+^ ions will be excited to the ^4^F_7/2_ energy state, and during this transition, some of the Er^3+^ ions will transit back to the ^4^I_15/2_ energy state, resulting in transition energy loss and small amounts of Er^3+^ ions will populate ^2^H_9/2_ energy state. Because of this energy loss, the gradient of emission at 410 nm is found to be less than 2, indicating that the transitions of weak violet UC emission may be caused by a two-photon mechanism [48,49].

The schematic energy level diagram of Er^3+^ and Yb^3+^ co-doped ZnTiO_3_ thin film is shown in Fig. 10(c). The ground state absorption (GSA) is known as the first transition for Yb^3+^ and Er^3+^ ions under 980 nm excitation. Since the absorption cross section of Yb^3+^ is larger than that of Er^3+^, the 980 nm excitation is mostly absorbed by the Yb^3+^ ions [50]. In the case of ZnTiO_3_:Er^3+^,Yb^3+^ thin films under an excitation of 980 nm, Yb^3+^ ions are excited from its fundamental ^2^F_7/2_ energy state and populate ^2^F_5/2_ energy state by absorbing 980 nm NIR photon via a GSA process. In this case, the non-radiative relaxation takes place from the ^2^F_5/2_ energy state back to ^2^F_7/2_ energy state and the ^4^I_11/2_, ^4^I_9/2_, ^2^F_7/2,_ and ^2^G_11/2_ energy states of Er^3+^ are populated through the following energy transfer from excited state of Yb^3+^ ions [[51], [52], [53]], Eq. (i) - [18,51], Eq. (ii) - [54,55], Eq. (iii) - [54,55], Eq. (iv) - [18,51] and Eq. (v) - [18,51].

[^4^I_13/2_ (Er^3+^), ^2^F_5/2_ (Yb^3+^)] → [^2^F_7/2_ (Yb^3+^), ^4^G_11/2_ (Er^3+^)] (i)

[^4^I_11/2_ (Er^3+^), ^2^F_5/2_ (Yb^3+^)] → [^2^F_7/2_ (Yb^3+^), ^4^F_7/2_ (Er^3+^)] (ii)

[^4^I_11/2_ (Er^3+^), ^2^F_5/2_ (Yb^3+^)] → [^2^_7/2_ (Yb^3+^), ^4^F_7/2_ (Er^3+^)] (iii)

[^4^I_11/2_ (Er^3+^), ^2^F_5/2_ (Yb^3+^)] → [^2^F_7/2_ (Yb^3+^), ^4^I_7/2_ (Er^3+^)] (iv)

[^4^I_13/2_ (Er^3+^), ^2^F_5/2_ (Yb^3+^)] → [^2^F_7/2_ (Yb^3+^), ^4^F_9/2_ (Er^3+^)] (v)

In addition, the ions at the ^4^G_11/2_ energy state of Er^3+^ depopulate to metastable ^2^H_9/2_, ^4^F_7/2_, ^4^S_3/2_, and ^4^F_9/2_ energy states through non-radiative transition while Er^3+^ ions in the ^2^F_7/2_ energy state are transferred non-radiatively to ^4^F_9/2_ energy state through a process called cross-relaxation (CR) energy transfer given by ^4^F_7/2_ → ^4^F_9/2_ and ^4^F_9/2_ ← ^4^I_11/2_ [14,56]. Eventually, the radiative decay of green and red emission due to ^2^H_9/2_ → ^4^I_15/2_, ^4^F_7/2_ → ^4^I_15/2_, ^4^S_3/2_ → ^4^I_15/2,_ and F_9/2_ → ^4^I_15/2_ transitions at 410, 480, 545 and 660 nm are observed.

**Fig. 11:**
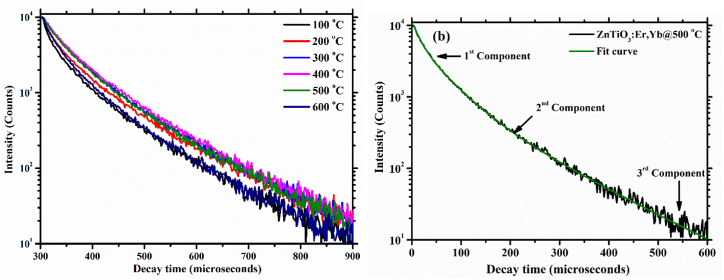
(a) and (b) Luminescence decay curves of ZnTiO_3_:Er^3+^,Yb^3+^ thin films at the emission centred at 545 nm.

Fig. 11 depicts the decay curves of the samples for emission band centred at 545 nm. With the increase in substrate temperature, the luminescence lifetime of the emission peak at 545 nm become faster up until a substrate temperature of 400 °C and then slower at a substrate temperature of 500 and 600 °C. Fig. 11(b) shows that the decay curves presented in 11(a) can be fitted with a third order exponential function and the average luminescence lifetimes can be estimated by the following equation (Eq. (2)) [56]

(2) $$\tau_{\text{avg}}=\frac{\int t\cdot I\left(t\right)\cdot dt}{\int I\left(t\right)\cdot dt}$$

where *I(t)* is the intensity at time *t*. The estimated average decay lifetimes of the thin films are summarized in Table 2.

**Table 2:** Photoluminescence lifetimes of ZnTiO_3_:Er^3+^,Yb^3+^ thin films.

Temperature (^o^C)	τ_1_(μs)	τ_2_(μs)	τ_3_(μs)	τ_avg_(μs)
100	113	486	1223	690
200	133	504	1283	805
300	297	635	1324	800
400	253	685	1402	842
500	218	629	1371	803
600	43	491	1228	688

## 4 Conclusion

ZnTiO_3_:Er^3+^,Yb^3+^ thin films were deposited at different substrate temperature on Si(100) using a PLD system. The surface roughness of the thin films decreased with an increase of the substrate temperature when the films were fabricated. Further increase in substrate temperature enriched the inter-diffusion process between Si and the ZnTiO_3_:Er^3+^,Yb^3+^ during the deposition, which decreased the thickness of the films and improved the luminescence properties of the samples. The SEM and TOF-SIMS results confirmed that Er^3+^ and Yb^3+^ ions were homogeneously distributed in the ZnTiO_3_ host lattice. The luminescence properties of the optimized ZnTiO_3_:Er^3+^,Yb^3+^ film which was deposited at a substrate temperature of 500 °C were superior as compared to other films. This was achieved by modifications in the electronic band structure ZnTiO_3_:Er^3+^,Yb^3+^ after heat treatments. Due to the UC transition of Er^3+^ paired with Yb^3+^ ions, the NIR photons were converted to visible light and can be utilized by ZnTiO_3_:Er, Yb thin films for application of UC materials. These studies revealed that the optimized film can be used as a promising candidate as an up-converter material.

## Author contribution statement

Sefako Mofokeng: Conceived and designed the experiments; Performed the experiments; Analyzed and interpreted the data; Wrote the paper.

Fokotsa V. Molefe, Teboho Patrick Mokoena, Makgamathe Joseph Sithole: Analyzed and interpreted the data; Wrote the paper.

Robin Edward Kroon, Hendrik C. Swart: Contributed reagents, materials, analysis tools or data; Wrote the paper.

Mkhotjwa S. Dhlamini, Luyanda L. Noto: Conceived and designed the experiments; Wrote the paper.

## Data availability statement

Data included in article/supplementary material/referenced in article.

## Declaration of competing interest

The authors declare that they have no known competing financial interests or personal relationships that could have appeared to influence the work reported in this paper
