# Supplementary material for: Substrate temperature dependence of structure and optical properties of ZnTiO3:Er3+/Yb3+ thin films synthesized by pulsed laser deposition
Source: Heliyon. 2023 May 12;9(5):e16259. doi: 10.1016/j.heliyon.2023.e16259 (PMC10205636; doi:10.1016/j.heliyon.2023.e16259)
Supplement: Multimedia component 1 [file mmc1.docx]

**Substrate temperature dependence of structure and optical properties of ZnTiO_3_:Er^3+^/Yb^3+^ thin films synthesized by pulsed laser deposition.**

S.J. Mofokeng^a*^_,_ F.V. Molefe^b^, R.E. Kroon^c^, H.C. Swart^c^, T.P. Mokoena^c^, M.S. Dhlamini^a^, S.J. Sithole^a^, L.L. Noto^a^,

^a^ Department of Physics, College of Science Engineering and Technology, University of South Africa, Johannesburg, 1710, South Africa

^b^ Department of Physics, Tshwane University of Technology, Private Bag X680, Pretoria 0001, South Africa  ^c^ Department of Physics, University of the Free State, Bloemfontein, ZA9300, South Africa

Corresponding author: [Sefakojmofokeng@gmail.com](mailto:Sefakojmofokeng@gmail.com)

**ELECTRONIC SUPPLEMENTARY INFORMATION**

A schematic representation of the fabrication of the thin films is shown in Figure S1.


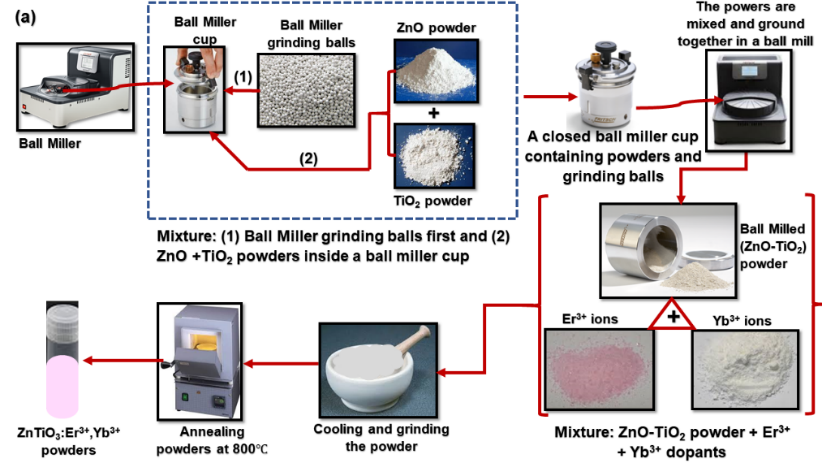


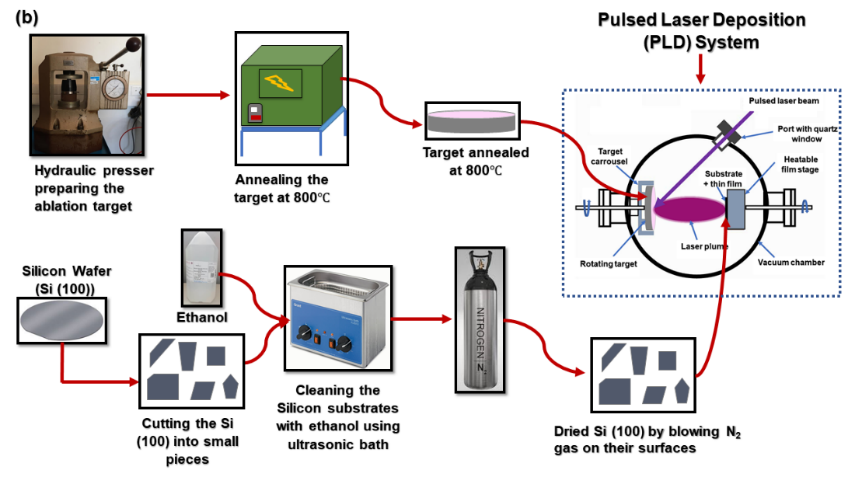


**Figure S1.** Schematic diagrams of the (a) experimental procedure of ZnO_3_:Er^3+^,Yb^3+^ powder prepared by conversional solid-state reaction technique and (b) fabrication of ZnO_3_:Er^3+^,Yb^3+^ thin films by a Pulsed Laser Deposition system (PLD).
